# Micro-Change Process of Calcium–Magnesium Double Expansive Agent and Its Performance Characterization in Cement-Based Materials

**DOI:** 10.3390/ma14123269

**Published:** 2021-06-13

**Authors:** Yihang Ding, Zhongyang Mao, Yu Wang, Min Deng, Mingshu Tang

**Affiliations:** 1 College of Materials Science and Engineering, Nanjing Tech University, Nanjing 211800, China; 201861203004@njtech.edu.cn (Y.D.); mzy@njtech.edu.cn (Z.M.); 201861203018@njtech.edu.cn (Y.W.); dengmin@njtech.edu.cn (M.D.); 2 State Key Laboratory of Materials-Oriented Chemical Engineering, Nanjing 211800, China

**Keywords:** cement-based materials, expansion agent, microstructure, performance

## Abstract

With the increase of cement output, the demand for cement expansion agents increases, and composite expansion agents have become the development trend. The purpose of this study is to study the microscopic change process and expansion effect of calcium oxide and magnesium oxide double expansion agents. After calcination at different temperatures, the change process of microscopic morphology of calcined products was observed. Through calcining dolomite at 900 °C, the mixture D900 of calcium oxide and magnesium oxide was obtained. To prepare mixed cement, 10 wt %, 20 wt %, and 30 wt % of D900 were added into cement to prepare mixed cement. At the same time, the compressive strength, deformation, and porosity of mixed cement were measured. The results show that adding D900 improves the expansion rate of early cement paste and reduces the compressive strength. After 120 days, the compressive strength of 20 wt % cement paste is higher than that of blank cement paste, and the porosity of 20 wt % cement paste is the lowest among the three mixed cements. This shows that 20 wt % is a more suitable substitute.

## 1. Introduction

Cement has a history of nearly 200 years since its birth and industrialization. As a result of its wide application range and large quantity, it has gradually become an indispensable and important substance in people’s production and life. In 2011, global cement production was 3.6 billion tons [1]. It is estimated that by 2050, global cement production will be 5.8 billion tons [2]. The increase of cement output will inevitably lead to the increase of the proportion of auxiliary cementitious materials used in concrete. Considering that the global annual output of fly ash and slag is 1 billion tons and 360 million tons respectively, such traditional auxiliary cementitious materials can only meet part of the demand of the concrete industry [3,4]. At present, concrete is the most widely used building material in the building field, but it often produces different types of damage before reaching the design life [5,6,7,8,9]. For various reasons, the shrinkage of cement-based materials is one of the key factors that leads to the instability of concrete volume, which in turn makes concrete crack and shortens its service life. Serious shrinkage cracking behavior may even lead to engineering accidents [9,10,11,12].

With the development of science and technology and the progress of society, people have gradually developed various types of expansive cement and concrete expansive agent to compensate for the shrinkage of concrete and improve its durability [13,14]. Concrete expansive agent is an additive developed on the basis of expansive cement, which can be mixed into concrete to produce concrete with expansive characteristics [15]. In the 1970s, China began to study magnesium oxide concrete and applied it to dams, water conservancy, and other large buildings. The expansion caused by the hydration of magnesium oxide in cement is used to compensate the temperature drop and shrinkage of mass concrete, which saves the project investment and accelerates the construction progress. With the development of magnesia concrete dam construction technology, magnesia expansive agent has been developed by calcining magnesite and added to concrete in the form of additives to prevent cracking and shrinkage [16,17]. In addition, magnesium oxide expansion agent can also be prepared by calcining magnesium-rich minerals such as dolomite and serpentine.

The theoretical chemical formula of dolomite is CaMg(CO_3_)_2_, which widely exists in nature and has a certain application basis in the field of construction. It is generally used as filler or aggregate. However, its application has been limited due to its high MgO content. The hydration reaction of dolomite in cement paste produces hydrated lime and brucite, and the expansion characteristics of this reaction will cause certain expansion stress, which will lead to cracking of hardened cement paste [18,19,20,21]. The final products of the complete decomposition of dolomite at high temperature are MgO and CaO [22]. The decomposition equation can be written as

CaMg(CO_3_)_2_ = MgO + CaO + CO_2_. (1)

In fact, the decomposition of dolomite takes place in two steps [23,24]. In the heating process of dolomite, MgO is firstly decomposed, and the decomposition equation is as follows.

CaMg(CO_3_)_2_ = MgO + CaCO_3_ + CO_2_ (2)

After that, the temperature continues to rise and CaCO_3_ is decomposed:

CaCO_3_ = CaO + CO_2_. (3)

By using the above characteristics and controlling the calcination temperature, completely decomposed dolomite can be obtained. This product can be considered as taking magnesium oxide and calcium oxide as the main mineral components, and both magnesium oxide and calcium oxide are the sources for preparing expansion agent. Therefore, the calcined product can be directly used as expansion agent. However, due to the irregular micro-morphology of the product, this experiment needs to observe and separate the micro-changes of Ca and Mg elements by changing the calcination system.

Light burnt dolomite is different from dead burnt dolomite, which shows high reactivity [23]. The addition of light burnt dolomite can release a large amount of expansion stress before the mud is completely hardened, thus avoiding the cracking of hardened mud due to expansion stress in the later period [24]. Therefore, this experiment requires two aspects of research. One is to prepare products with different product components by changing the calcination system. Since MgO crystals are endothermic and grow due to the increase in temperature, it can be distinguished by the morphology of the product after calcination at a lower temperature and the morphology of the product after calcination at high temperature. The second aspect is to mix the MgO-CaO product produced by calcining dolomite into cement in different proportions to prepare mixed cement and analyze the feasibility of the calcium–magnesium double expansion agent by comparing the change of pore structure and the deformation performance.

## 2. Materials and Methods

### 2.1. Raw Materials

In this study, 52.5 Portland cement was used alongside the dolomite from Chizhou, Anhui Province. The chemical composition of cement and dolomite was tested according to GB/T 176-2017 (CIS, 2017), as shown in Table 1. Dolomite was calcined at 900 °C for 1 h to produce D900, with calcite and calcium oxide as the main components. After cooling, it was ground into powder of less than 80 μm. With cement as the raw material, D900 as additive, and a water–cement ratio of 0.28, rectangular cement paste samples of 20 mm × 20 mm × 80 mm were prepared by the internal mixing method. Several groups of samples were placed in distilled water solution and solidified at room temperature of 20 °C to obtain samples of different ages.

D900 was partially substituted for PC (10%, 20% and 30% by mass) to prepare cement paste samples. The cement paste mix ratio design is shown in Table 2.

### 2.2. The Experimental Methods

#### 2.2.1. XRD Analysis

The dolomite products of different calcination temperatures and cement paste samples of different ages were analyzed by X-ray diffraction. The experimental powder should pass through a 0.08 mm screen. XRD data were collected at 5°–80°, 2θ at a count time of 15 s/step and divergent slit of 1°.

#### 2.2.2. Thermogravimetric (TG)—Differential Scanning Calorimetry (DSC) Analysis

The TG-DSC data of cement were obtained in the range of 50 to 900 °C at a heating rate of 10 °C/min in N_2_ atmosphere. The final products of dolomite decomposition are calcite and calcium oxide. The reaction is as follows:

CaMg(CO_3_)_2_ = MgO + CaO + CO_2_. (4)

#### 2.2.3. SEM Analysis

In order to study the microstructure of dolomite products, the surface morphology of dolomite products was observed by scanning electron microscope (JSM 6510, Jeonju, Tokyo, Japan).

#### 2.2.4. Determination of the Heat of Hydration of Cement

The principle of hydration heat measurement is based on the Gass law of thermochemistry. The exotherm of a chemical reaction is only related to the initial and final state of the system, not to the reaction process. Under the condition of a certain temperature around the calorimeter, calcium oxide and magnesium oxide dissolve in water, and hydration reaction occurs. The heat released within a certain period of time is called the heat of hydration.

#### 2.2.5. Test of Expansion Property of Cement Paste Specimen

The deformation properties of cement paste specimens of different ages were tested. According to Equation (5), at a certain age, the length of the sample is measured with a specific length meter with an accuracy of 0.01%, and the expansion of the sample is calculated by taking the average value of the measured data. In the formula, *L*_0_ represents the initial length, *L* represents the effective length of 80 mm, and *L_t_* represents the length of pure paste specimens at different ages.

(5) $$P=\frac{Lt-L_{0}}{L}\times 100\%$$

#### 2.2.6. Test for Compressive Strength of Cement Paste

The compressive strength of cement paste at different ages was tested by using a compression testing machine with a loading rate of 0.5–0.8 MPa/s. The average compressive strength of the three cement paste samples was used for each test result.

#### 2.2.7. Pore Structure of Cement Paste

Mercury intrusion porosimetry (MIP) is a method that can accurately measure the porosity and pore size distribution of cement materials. In this study, we used molecularly imprinted polymer (AutoPore W9500, Micromeritics, Norcross, GE, USA) to characterize the porosity and pore distribution of samples, and we analyzed the corrosion process of materials from the pore structure.

## 3. Results and Discussion

### 3.1. Microscopic Characterization of Expansive Agent

#### 3.1.1. XRD Analysis of Expansive Agent

**Figure 1:**
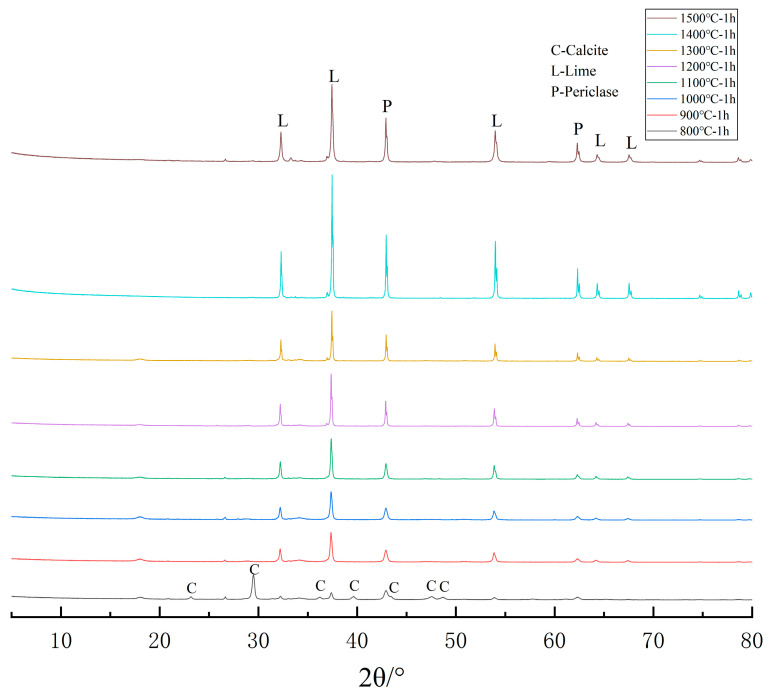
XRD pattern of calcined products of dolomite.

Figure 1 is an XRD pattern of different calcination temperatures for 1 h. It can be seen from the diffraction peaks in Figure 1 that dolomite is decomposed into CaCO_3_, MgO, and a small amount of CaO after being kept at 800 °C for 1 h, and it completely decomposed into CaO and MgO after being kept at 900 °C for 1 h. When the temperature gradually increased to 1500 °C and was kept for 1 h, the composition of the product did not change. The results show that after 900 °C, the increase of calcination temperature will not lead to the change of product composition, so the micro-morphology change of the product can be observed in the temperature range from 900 to 1500 °C. However, as the increase of calcination temperature will reduce the activity of the product, the suitable temperature for testing the expansion property is 900 °C for 1 h.

#### 3.1.2. The SEM Analysis of Expansive Agent

**Figure 2:**
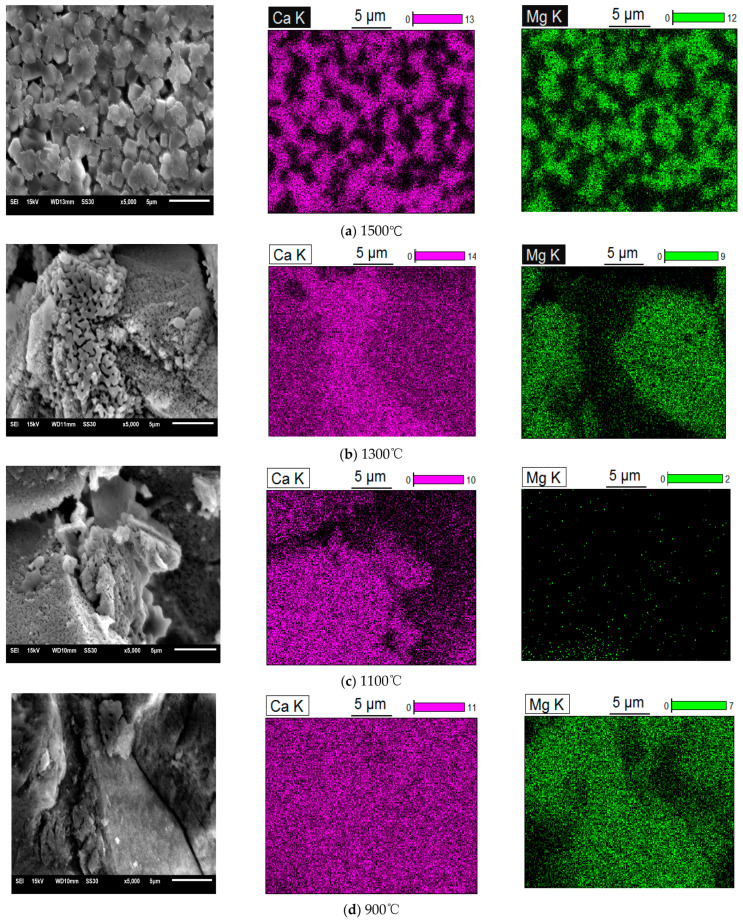
SEM images of dolomite products held at different calcination temperatures for 1 h.

Figure 2 shows the microscopic morphology of dolomite calcined at different temperatures. Figure 2a is an SEM image of dolomite calcined at 1500 °C for 1 h. It can be seen from the element distribution map that the petal-shaped particles are Ca elements and the regular particles are Mg elements. It can be seen that when dolomite is calcined at 1500 °C for 1 h, calcium oxide and magnesium oxide in the product are separated to form clear morphological characteristics, but they still adsorb each other.

Figure 2b is an SEM image of dolomite calcined at 1300 °C for 1 h. It can be seen from the element distribution diagram that the strip product is a Ca element. Therefore, when dolomite is calcined at 1300 °C for 1 h, part of CaO in the product is separated into strip products, while other CaO and MgO still form a block mixture.

Figure 2c is an SEM image of dolomite calcined at 1100 °C for 1 h. It can be seen from the element distribution diagram that the surface of this area is almost full of calcium, but because dolomite products also contain a large amount of magnesium oxide, it can be seen from the elemental analysis on the diagram that calcium is wrapped on the surface of magnesium in the products kept at 1100 °C for 1 h.

Figure 2d is an SEM image of dolomite after being kept at 900 °C for 1 h. It can be seen from the element distribution map that almost all areas are blocky products formed by mixing Ca and Mg elements, and there is no separation and coating phenomenon.

It can be seen from the above SEM images that magnesium oxide and calcium oxide in dolomite products exist as a mixture when calcined at 900 °C and 1100 °C for 1 h. When the temperature rises to 1300 °C for 1 h, some calcium elements are separated from the mixed state and form regular strip crystals, while some calcium elements and magnesium elements remain in the mixed state. When the temperature rises to 1500 °C for 1 h, calcium and magnesium are completely separated, and calcium exists as petal-shaped crystals, while magnesium exists as blocky crystals. The results show that calcium oxide and magnesium oxide exist in the form of a mixture in the product of dolomite calcination temperature below 1300 °C.

### 3.2. Performance Characterization of Mixed Cement

#### 3.2.1. Hydration Heat Curve of Mixed Cement

**Figure 3:**
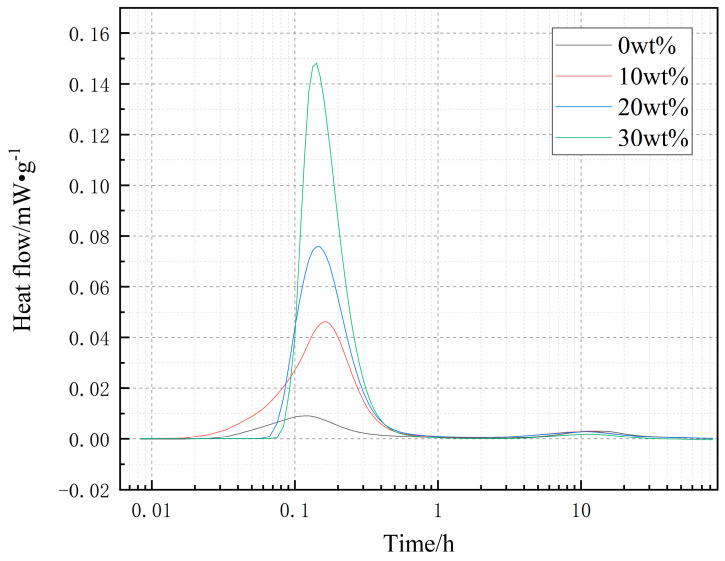
Heat of hydration curve of cement with different dosage.

Figure 3 is a graph of hydration heat with different dosages. It can be seen from the figure that there is a large exothermic peak after stirring for 0.1 h, which is due to the fact that CaO in D900 will release a large amount of heat when contacting with water, forming an exothermic peak. With the increase of D900 content, the more heat is released. After stirring for 10 h, MgO undergoes a hydration reaction and gives off heat, but the hydration heat of MgO is much lower than that of CaO, so the hydration heat release peak of MgO in the figure is not obvious. The cumulative heat release of mixed cement with 0 wt %, 10 wt %, 20 wt %, and 30 wt % D900 for 3 days is 134.24 kJ, 290.49 kJ, 294.65 kJ, and 306.26 kJ, respectively.

#### 3.2.2. Expansion Rate Change Diagram of Mixed Cement

**Figure 4:**
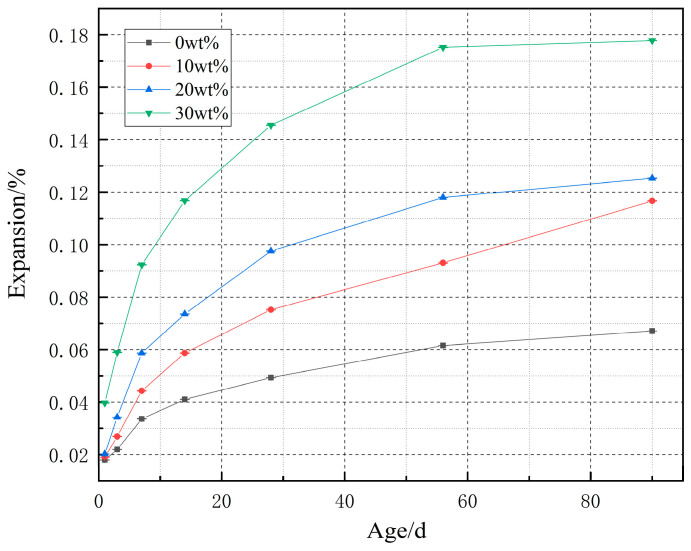
Expansion change data of pure paste samples with different dosage.

Figure 4 is a data diagram of the expansion rate change of clean pulp samples with different dosages. It can be seen from the figure that the swelling rates of cement paste samples with four dosage change the most within 7 days, and the swelling rates are 0.0336%, 0.0443%, 0.0587%, and 0.0923%, respectively. After 14 days, the expansion of the cement blank tends to be flat. After 28 days, the cement with 10 wt %, 20 wt %, and 30 wt % content expanded continuously, and the main source of expansion was the hydration reaction of MgO, which produced an expansion force. With the increase of content, the expansion rate of the cement increased. At the end of 90 days, the swelling rates of cement paste samples with four dosage levels were 0.0671%, 0.1167%, 0.1253%, and 0.1778%, respectively. After 90 days, for the samples containing 10 wt %, 20 wt %, and 30 wt % cement paste, the expansion changes tend to be flat. At the end of 180 days, the swelling rates of cement pastes with four kinds of content are 0.0704%, 0.1358%, 0.1452%, and 0.1981%, respectively.

#### 3.2.3. Change Diagram of Compressive Strength of Mixed Cement

**Figure 5:**
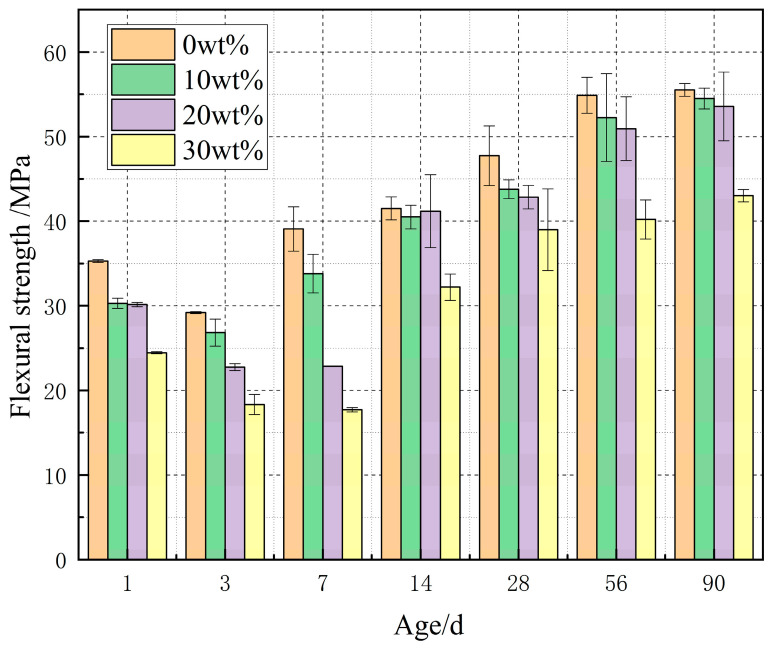
Compression change data graph of pure paste specimens with different dosages.

Figure 5 is a data diagram of compressive strength change of clean pulp samples with different dosage. The compressive strength of cement paste specimens with four dosages at the end of one day is 35.29385 MPa, 30.2826 MPa, 30.13975 MPa, and 24.4557 MPa, respectively. With the increase of dosage, the compressive strength decreases gradually. The compressive strength of specimens decreased obviously at the end of 3 days. At the end of 7 days, the strength of the blank samples and cement paste samples with 10 wt % content increased to some extent, and then, the compressive strength increased with the longer curing time. However, the strength of specimens with 20 wt % and 30 wt % cement paste is still decreasing at the end of 7 days, and the compressive strength of specimens increases obviously after the curing time reaches 14 days, and the compressive strength of specimens with 20 wt % cement paste is almost the same as that with 10 wt % cement paste at the end of 14 days to 90 days. At the end of 120 days, the strength of the blank sample and cement paste samples with 10 wt % and 20 wt % decreased by −11.80%, −15.20%, and −7.86%, respectively compared with that at the end of 90 days, while the strength of cement paste samples with 30 wt % increased by 6.65%. At this time, the strength of the specimen mixed with 20% cement paste is higher than that of other specimens, and at the end of 180 days, the strength of the specimen mixed with 4 doses of cement paste decreases relatively. According to the late results of Figure 4 and Figure 5, 20 wt % is suitable.

#### 3.2.4. TG-DSC Spectra of Mixed Cement in Different Impending Periods

**Figure 6:**
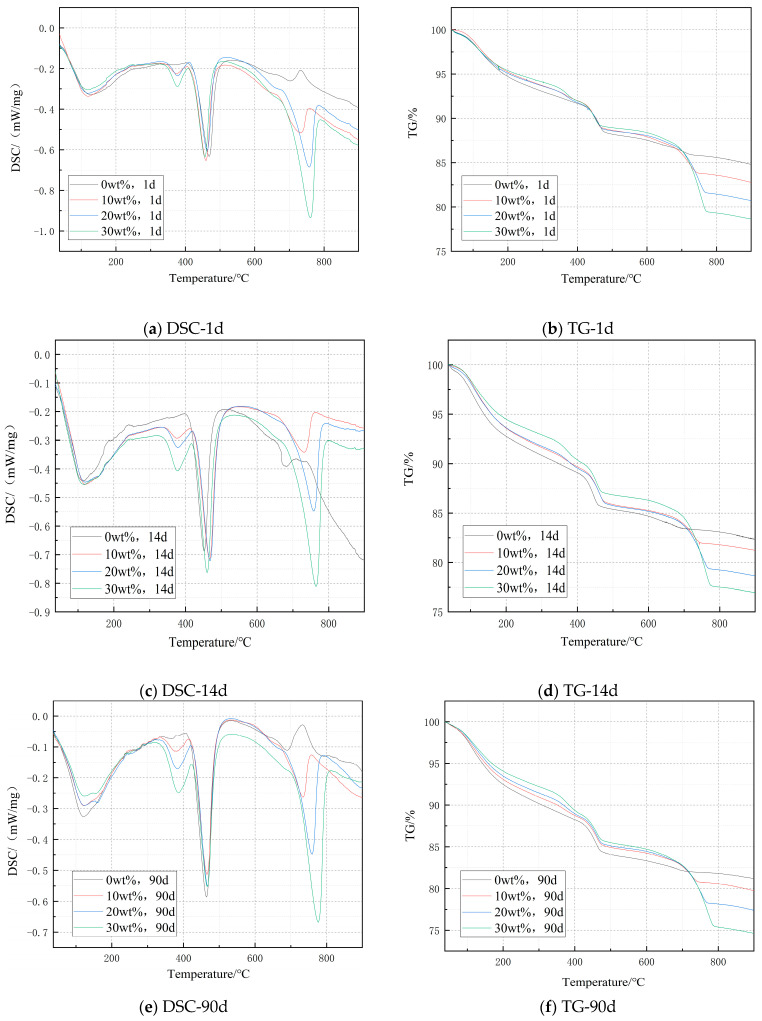
DSC-TG diagram of cement paste specimens with different dosages after curing.

Figure 6 shows the DSC and TG spectra of cement paste specimens with different dosages after curing. It can be seen from the DSC curve in the figure that there are three obvious endothermic peaks in the temperature ranges of 350–400 °C, 400–500 °C, and 650–800 °C, which are caused by the thermal decomposition of Mg(OH)_2_, Ca(OH)_2_, and CaCO_3_. In the temperature range of 650–800 °C, the content of CaCO_3_ in the cement increases with the increase of curing time and dosage, which leads to the increase of endothermic peak, and it makes the complete decomposition temperature of CaCO_3_ gradually approach 800 °C.

According to the TG curves shown in Figure 6, the mass loss of Mg(OH)_2_ at this temperature can be known, and the amount of Mg(OH)_2_ formed in cement paste and the hydration degree of MgO can be calculated by Equations (6) and (7).

(6) $${\mathrm{Mass}}_{\mathrm{Mg}{\left(\mathrm{OH}\right)}_{2}}=\frac{58\times \mathrm{Mass}\;{\mathrm{loss}}_{\left(320{}^{\circ}C\text{-}400{}^{\circ}C\right)}}{18}$$

(7) $${\mathrm{Hd}}_{\mathrm{MgO}}=\frac{40\times \mathrm{Mass}\;{\mathrm{loss}}_{\left(320{}^{\circ}C\text{-}400{}^{\circ}C\right)}}{0.08\times 18\times \left[1-\mathrm{Mass}\;{\mathrm{loss}}_{\left(950{}^{\circ}C\right)}\right]}$$

In the formula, ${\mathrm{Mass}}_{{\mathrm{Mg}}_{{\left(\mathrm{OH}\right)}_{2}}}$ and ${\mathrm{Hd}}_{\mathrm{MgO}}$ respectively represent the amount of Mg(OH)_2_ formed in the paste and the hydration degree of MgO. Mass loss_(320__°C–400__°C)_ refers to the mass loss caused by the decomposition of Mg(OH)_2_ in cement paste at 320–400 °C or the mass loss caused by the blank sample of cement paste within this temperature range. Mass loss_(950__°C)_ indicates the mass loss of cement paste hardened at 950 °C.

Table 3 shows the amount of Mg(OH)_2_ and hydration degree of MgO in cement paste. Obviously, with the development of curing time and the increase of D900 content, the content of Mg(OH)_2_ increased obviously. See Table 3 for the Mg(OH)_2_ content and hydration degree of MgO in cement paste.

#### 3.2.5. Porosity Change Diagram of Mixed Cement in Different Impending Periods

**Figure 7:**
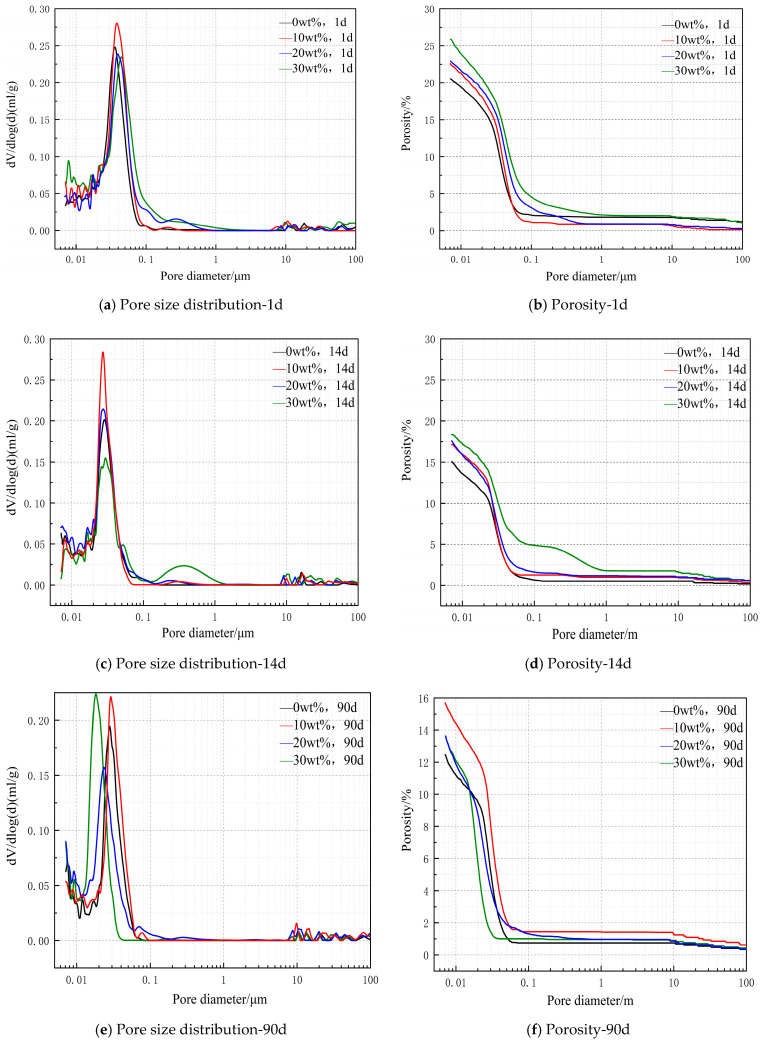
Pore size distribution and cumulative porosity of cement paste samples with different dosages after curing.

Figure 7 shows the pore size distribution and cumulative porosity of cement paste samples with different dosages after curing at room temperature. Figure 7a,b show the pore size distribution and cumulative porosity of cement pastes with different dosages after curing for 1 day. As the hydration reaction of CaO occurred in the early stage, more holes were produced in the pure paste samples at the end of one day, and the pore diameter was mainly concentrated in 0.02–0.08 μm diameter. The pore structure of cement paste samples without dolomite products is dense, and the cumulative porosity of cement paste samples with 10 wt %, 20 wt %, and 30 wt % dolomite or no dolomite is 22.5842%, 22.9001%, 25.8545%, and 20.5295%, respectively.

Figure 7c,d show the pore size distribution and cumulative porosity of cement pastes with different dosages after curing for 14 days. At the end of 14 days, there are many holes in the clean pulp sample, and the pore size is mainly concentrated in 0.02–0.08 μm diameter. There are many holes with diameters of 0.1–1.1 μm in 30 wt % cement paste, which is caused by high calcium oxide content. The cumulative porosity of cement paste containing 10 wt %, 20 wt %, and 30 wt % dolomite or no dolomite products is 17.1345%, 17.6195%, 18.3265%, and 15.1255%, respectively.

Figure 7e,f show the pore size distribution and cumulative porosity of cement paste with different dosages after curing for 90 days. At the end of 90 days, there were many pores in the pure pulp specimen, and the pore diameter was mainly concentrated in 0.01–0.06 μm diameter. There are many holes with diameters of 0.01–0.04 μm in 30 wt % cement paste, which is caused by the high content of calcium oxide. The pore structure of cement paste samples without dolomite products is relatively dense, and the cumulative porosity of cement paste with 10 wt %, 20 wt %, and 30 wt % dolomite or without dolomite products is 15.6971%, 13.6159%, 13.6195%, and 12.4684%, respectively. Compared with the 14-day data chart, the pores of cement paste with 10 wt %, 20 wt %, and 30 wt % content are obviously smaller, which is due to the hydration reaction of MgO, which produces a certain expansion force and plays a certain role in improving the internal pore structure.

### 3.3. Discussion

It can be seen from the XRD analysis chart of Figure 1 and the SEM image of Figure 2 that when the calcination temperature is higher than 900 °C, the composition of the dolomite calcined product will not change with the increase of temperature. However, due to the increase of the calcination temperature, the morphology of the components in the product will change. Therefore, it can be seen that the microscopic morphology of the dolomite product calcined at 900 °C is not the mixture form in the SEM image.

Xu [24] reported that the calcined dolomite product contains CaO. CaO will become an interference factor for cement. Therefore, the calcined product of complete decomposition of dolomite cannot be directly added to cement for use. SiO_2_ is needed to consume CaO during the calcination process. From the results of my experimental data, it is feasible to add the 20 wt % dolomite calcined product to cement. The early strength of 20 wt % cement is lower than that of the blank cement group, and the late strength is higher than that of the blank cement group. The expansion rate of 20 wt % cement is relatively good. The calcined dolomite produces a certain expansion in the cement, improves the pores in the cement, and increases the compressive strength of the cement paste. Therefore, I think that dolomite calcined products can be used directly to replace part of the cement to prepare mixed cement.

## 4. Conclusions

The main conclusions are as follows:

I. In the SEM images of dolomite calcined products, when the calcination temperature rises to 1300 °C, some calcium elements are separated from the mixture to form regular strip crystals, while magnesium elements have no obvious morphological characteristics. When the calcination temperature rises to 1500 °C, calcium and magnesium are completely separated, and petal-shaped calcium and block-shaped magnesium are adsorbed to each other, forming an irregular structure. This shows that MgO and CaO produced by dolomite calcined at 900 °C exist in the form of a mixture, and the existing structure will change with the increase of temperature until they separate from each other to form a single crystal.

II. Before 90 days, the swelling rate increased with the increase of dosage. After 90 days, the expansion rates of blank cement paste specimens and cement paste specimens containing 10 wt %, 20 wt %, and 30 wt % D900 are 6.711%, 11.667%, 12.833%, and 18.578%, respectively. After 90 days, the expansion rate of cement paste samples with different content tends to be flat. The compressive strength of cement paste samples with 10 wt %, 20 wt %, and 30 wt % D900 before 90 days is lower than that of the samples without cement paste, and with the increase of the content, the compressive strength decreases more. At the end of 120 days, the strength of the specimen with 20 wt % cement paste is higher than that without cement paste, which is due to the expansion stress produced by a MgO hydration reaction. According to the expansion effect and the change of compressive strength, 20 wt % is the most suitable mixing ratio.

III. When D900 is mixed with ordinary Portland cement, the early heat release of cement paste samples is obvious, and the number of pores in cement paste increases. With the increase of the content, the number of pores in the cement paste increases, and the curing time is prolonged, which makes the pore diameter of the cement paste become obviously thinner. Porosity decreases with the increase of curing time. The porosity of 90-day blank cement paste specimens and cement paste specimens doped with 10 wt %, 20 wt %, and 30 wt % D900 are 12.4684%, 15.6971%, 13.6158%, and 13.6195%. This shows that MgO hydration produces expansive force in 90 days, which has a certain effect on improving the internal pore structure.

## Figures and Tables

**Table 1:** Chemical composition of Portland cement clinker and dolomite.

Type	LOI *	SiO_2_	CaO	MgO	Fe_2_O_3_	Al_2_O_3_	SO_3_
Dolomite	45.68	0.98	30.89	20.66	0.24	0.68	-
Cement	2.76	19.29	64.81	2.33	3.47	4.89	1.57

* Loss on ignition.

**Table 2:** Mix proportions of concrete samples.

Samples	Water	Binder	
		PC	D900
PC	126	450	0
10 wt %	126	405	45
20 wt %	126	360	90
30 wt %	126	315	135

**Table 3:** Estimated quantity of Mg(OH)_2_ and hydration degree of MgO.

Samples	Ref.			10 wt %			20 wt %			30 wt %		
	1d	14d	90d	1d	14d	90d	1d	14d	90d	1d	14d	90d
Mass loss at 320–400 °C/wt %	1.0	1.3	1.7	1.4	1.8	2.1	1.8	2.0	2.6	2.0	2.6	3.4
Estimated quantity of Mg(OH)_2_	3.2	4.2	5.5	4.5	5.8	6.8	5.8	6.4	8.4	6.4	8.4	11.0
Estimated hydration degree of MgO	34.9	45.4	59.3	48.8	62.8	73.3	62.8	69.8	90.7	69.8	90.7	118.6

## Data Availability

The data presented in this study are available on request from the corresponding author.

## References

[1] Cembureau (2011). European 2010 production down. Int. Cement Rev..

[2] Scrivener K. (2012). Issues in sustainability in cements and concrete. Am. Ceram. Soc. Bull..

[3] Malhotra V.M. (2006). Reducing CO_2_ emissions-the role of fly ash and other supplementary cementitious materials. Concr. Int..

[4] Reynolds S. (2009). The Future of Ferrous Slag-Market Forecasts to 2020.

[5] Tang M.S. (2007). Service-life of Infrastructure Engineering to be Emphasized in Energy Conservation and Emission Reduction. China Cement.

[6] Aitcin P.C. (2000). Cements of yesterday and today Concrete of tomorrow. Cem. Concr. Res..

[7] Zhang W., Yang Q.B. (2003). Review on shrinkage of concrete. Low Temp. Arch. Technol..

[8] Mo L.W., Deng M. (2010). Review on the resarch of MgO-based expansive agent. Expans. Agents Expans. Concr..

[9] Chatterji S. (1995). Mechanism of expansion of concrete due to the presence of dead-burned CaO and MgO. Cem. Concr. Res..

[10] Chatterji S. (2005). Aspects of generation of destructive crystal growth pressure. J. Cryst. Growth.

[11] Metha P.K. (1982). Expansion of ettringite by water adsorption. Cem. Concr. Res..

[12] Mo L., Liu M., Al-Tabbaa A., Deng M., Lau W.Y. (2015). Deformation and mechanical properties of quaternary blended cements containing ground granulated blast furnace slag, fly ash and magnesia. Cem. Concr. Res..

[13] You B.K., Qi D.Y. (2014). Comments on MgO expensive agent. Expans. Agent Expans. Concr..

[14] Zhang G., Li G. (2016). Effects of mineral admixtures and additional gypsum on the expansion performance of sulphoaluminate expansive agent at simulation of mass concrete environment. Constr. Build. Mater..

[15] Meng G., Zhang K.F. (2013). Research Progress of Expansive Agent in Expansive Concrete. Mater. Rev..

[16] Li C.M., Yuan M.D. (2003). A summary of applications of dam construction technology with MgO-mixed micro-expansion concrete. Adv. Sci. Technol. Water Resour..

[17] Li C.M. (2013). Review of quick damming technology of MgO concrete. Adv. Sci. Technol. Water Resour..

[18] Gabrovsek R., Vuk T., Kaucic V. (2006). Evaluation of the hydration of Portland cement containing various carbonates by means of thermal analysis. Acta Chim. Slov..

[19] Galí S., Ayora C., Alfonso P., Tauler E., Labrador M. (2001). Kinetics of dolomite-portlandite reaction. Application to Portland cement concrete. Cem. Concr. Res..

[20] Taylor H.F.W. (1990). Cement Chemistry.

[21] Tong L., Tang M. (1999). Expansion mechanism of alkali-dolomite and alkali-magnesite reaction. Cem. Concr. Compos..

[22] Xu L.L., Deng M. (2005). Dolomite used as raw material to produce MgO-based expansive agent. Cem. Concr. Res..

[23] Geng F., Gao P.W., Luo J.J. (2012). Influence of Calcination Temperature on Expansive Performance of a Novel Ecological Expansive Agent. J. Anhui Univ. Technol..

[24] Deng Y., Deng M., Mo L.W. (2012). Influence of periclase contained in cement clinker and lightly-burnt MgO-based expansive agent on expansion of cement pastes. Concrete.

